# Blood flow restricted exercise with and without transcutaneous spinal cord stimulation via telehealth in persons with tetraplegia. A protocol for randomized clinical trial

**DOI:** 10.1038/s41393-026-01275-1

**Published:** 2026-09-19

**Authors:** Ashraf S. Gorgey, Muhammad U. Rehman, Momal Wasim, Refka E. Khalil, Bret Brooks, Ronald Seel, Rajbir Chaggar, Timothy Lavis

**Affiliations:** 1 Spinal Cord Injury and Disorders, Richmond VA Medical Center, Richmond, VA USA; 2 Virginia Commonwealth University, Department of Physical Medicine & Rehabilitation, Richmond, VA USA; 3 Virginia Commonwealth University, Department of Biomedical Engineering, Richmond, VA USA; 4 Virginia Commonwealth University; Center for Rehabilitation Science and Engineering (CERSE), Richmond, VA USA

**Keywords:** Randomized controlled trials, Spinal cord diseases

## Abstract

**Study design:**

A single site randomized clinical trial; Richmond, VA, USA.

**Objective:**

To determine the effect of 16 weeks of home-based blood flow restriction-enhanced neuromuscular electrical stimulation (BES) and non-invasive transcutaneous spinal cord stimulation (TSS) with mass practiced (MP) training on cervical neuroplasticity and upper extremity function in individuals with tetraplegia. The program is supported by a telehealth-based approach designed to improve adherence.

**Methods:**

Forty-four adults (18–70 years) with incomplete cervical SCI (AIS B–D; injury level C8 or above) will be randomized to TSS + BES + MP or *sham* TSS + BES + MP for 30 weeks. All participants, accompanied by a caregiver, will complete a 4-week education and training phase (2 weeks on-site, 2 weeks via telehealth), followed by 16 weeks of telehealth-supervised home training. Baseline assessments will occur before training (BL1) and after the education phase (Phase 1, BL2). Post-intervention (P1) testing will follow 16 weeks of home-based training (Phase 2), with an additional follow-up (Fw) after an 8-week washout period to evaluate any carry-over effects (Phase 3). The first 15 months will serve as a feasibility phase without randomization; data from these participants will be included within the TSS + BES + MP group. Randomization thereafter will be conducted using a computer-generated sequence.

**Safety and Summary:**

Home-based TSS application for upper extremity function is a feasible and safe strategy in persons with tetraplegia. The proposal is likely to ensure long-term compliance and adherence as well as to reduce the barriers associated with exercise interventions in persons with tetraplegia.

**Sponsorship:**

National Institute on Disability, Independent Living, and Rehabilitation Research [NIDILRR].

**ClinicalTrials.gov Identifier:**

NCT05423600.

**Figure:**
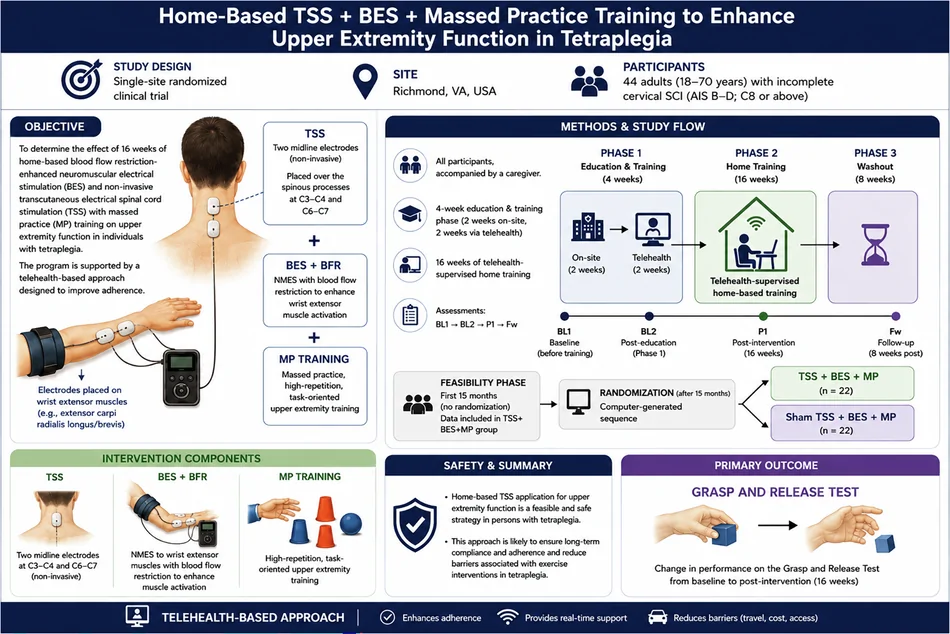

## Introduction

Tetraplegia causes severe motor impairment, loss of upper-extremity and trunk control, and loss of independence in daily activities. Secondary cardiovascular, respiratory, metabolic, and psychosocial complications further increase morbidity and socioeconomic burden [1–3]. Although advances in acute care have improved survival, lifelong medical costs continue to escalate and impact quality of life (QOL) [4]. Restoration of arm and hand function remains a top rehabilitation priority, as greater dexterity directly enhances independence and QOL [5]. While epidural stimulation can facilitate motor recovery [6], its invasiveness limits widespread use, underscoring the need for noninvasive, scalable alternatives.

Transcutaneous spinal cord stimulation (TSS) is a promising neuromodulatory technique that activates spinal networks supporting motor and respiratory functions [7–11]. TSS enhances electromyographic activity, reduces reflex excitability, and improves limb movement without adverse effects [12–14]. When combined with massed practice (MP) repetitive task-specific exercise, TSS may induce cortical and spinal neuroplasticity that improves upper-extremity control [15, 16]. Previously, we noted that blood-flow-restriction-enhanced neuromuscular electrical stimulation (BES) increases muscle size and strength compared to the contralateral upper extremity following conventional neuromuscular electrical stimulation (NMES) in persons with chronic incomplete SCI [17]. This was accompanied by improvement in hand functions as measured by the grasp and release test [17]. Therefore, pairing BES with TSS may synergistically promote neuroplastic and muscular adaptations by recruiting afferent fibers that may further augment cervical neural excitability and enhance upper extremity function.

The current study summarizes the study protocol of a telehealth-supported, home-based TSS + BES + MP intervention for individuals with chronic, incomplete tetraplegia. The cardiovascular safety and feasibility of the proposed home-based TSS + BES will also be determined to ensure safety against development of autonomic dysreflexia and practicality of allowing caregivers to administer the proposed therapy without development of any adverse events. We hypothesize that TSS + BES + MP will elicit greater improvements in upper-extremity strength, electromyography (EMG) activation, and functional performance compared with sham TSS + BES + MP. The study will also explore the potential of enhancing pulmonary and cardiovascular-autonomic outcomes [7, 18].

## Methods

### Study design

**Fig. 1:**
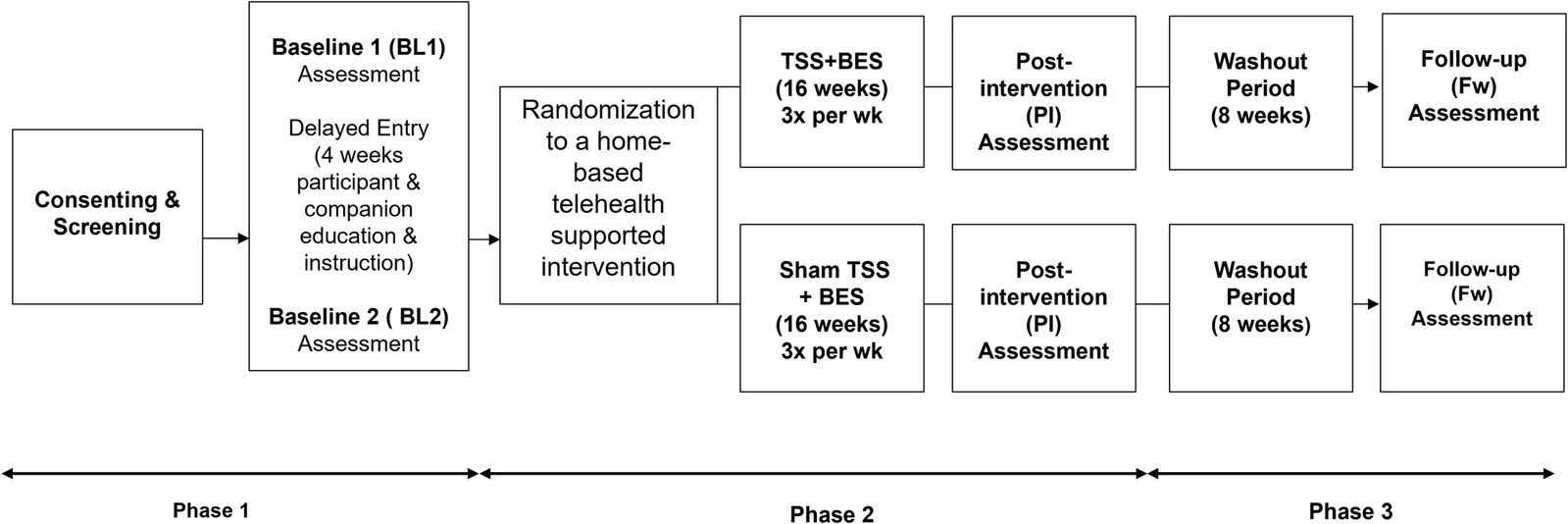
Trial timeline and assessment schedule. Participants complete consenting/screening, Baseline 1 (BL1), and a 4-week delayed-entry education phase in which the participant and companion are trained on device setup and safety, followed by Baseline 2 (BL2). After BL2, participants are randomized to TSS + BES + massed practice (MP) or sham TSS + BES + MP for 16 weeks of largely telehealth-supervised home training, after which a post-intervention (PI) assessment is performed. An 8-week washout period follows, ending with a follow-up (Fw) assessment to evaluate carry-over and retention. TSS, transcutaneous spinal cord stimulation; BES, blood-flow-restriction–enhanced neuromuscular electrical stimulation; MP, massed practice; BL1/BL2, Baseline 1/2; PI, post-intervention; Fw, follow-up.

As outlined in Fig. 1, forty-four adults (18–70 years) with chronic, incomplete cervical SCI (AIS B–D) and a caregiver or companion will complete a 4-week education phase 1 (2 weeks onsite, 2 weeks via telehealth) followed by 16 weeks of supervised-telehealth training (Phase 2; TSS + BES + MP versus sham TSS + BES + MP). The neurological level of injury will be determined using the ASIA/ISCoS International Standards for Neurological Classification of Spinal Cord Injury (ISNCSCI).

Baseline assessments will occur before education (BL1) and after the 4-week training phase (Phase 1, BL2); post-intervention testing (PI) will follow 16 weeks of home-based training (Phase 2), and a follow-up (Fw) will be conducted after an 8-week washout period to evaluate any carry-over effects (Phase 3). The first 15 months will serve as a feasibility phase without randomization.

### Screening and consenting

All eligible participants will provide written consent and undergo medical screening by an SCI physician, including physical examination, electrocardiogram (ECG), oxygen saturation, and pulmonary function testing. Questionnaires assessing health, wellness, and quality of life will be administered at BL1, BL2, PI, and Fw. Participants and caregivers will receive structured instructions on how to effectively use the stimulator, BES and MP exercises, and telehealth procedures, while stimulation parameters are optimized for efficacy and comfort. All methods and procedures were approved by the Richmond VA Medical Center (Protocol # 664373) and Virginia Commonwealth University (IRB HM20023453) local IRBs prior to initiation of the study. The clinical trial was registered at clinicaltrials.gov (NCT05423600). Finally, all methods will be performed in accordance with the relevant guidelines and regulations. Informed consent will be obtained from all participants prior to starting any study procedures. The consent form includes a clear statement about capturing photographs for the purpose of research, education and sharing knowledge. Each participant has to consent that his/her photographs are captured during research visits.

### Randomization and allocation

In the first 15 months, recruited participants will only be enrolled in a feasibility portion of the trial and will be treated as a part of the TSS + BES + MP group. Randomization will be conducted following the baseline assessment periods using random number generator program. Each participant will be assigned a sequential ID code [006-044].

### Measurements

Heart rate and blood pressure will be continuously monitored during all on-site education procedures (Phase 1; first 4 weeks of the trial) and before and after each home-based training session with TSS off (Phase 2; following 16 weeks). Participants and caregivers will be trained to measure blood pressure using a portable digital brachial or wrist monitor units. Despite previous accuracy reports [19], wrist units are allowed to ensure simplicity of measuring BP in persons with chronic tetraplegia. If systolic pressure rises >20 mmHg above baseline or autonomic dysreflexia (AD) symptoms occur, the session will be ceased immediately, the participant will rest, and study staff and medical personnel will be notified for further evaluation [18].

#### Primary outcomes

##### Grasp and release test

**Fig. 2:**
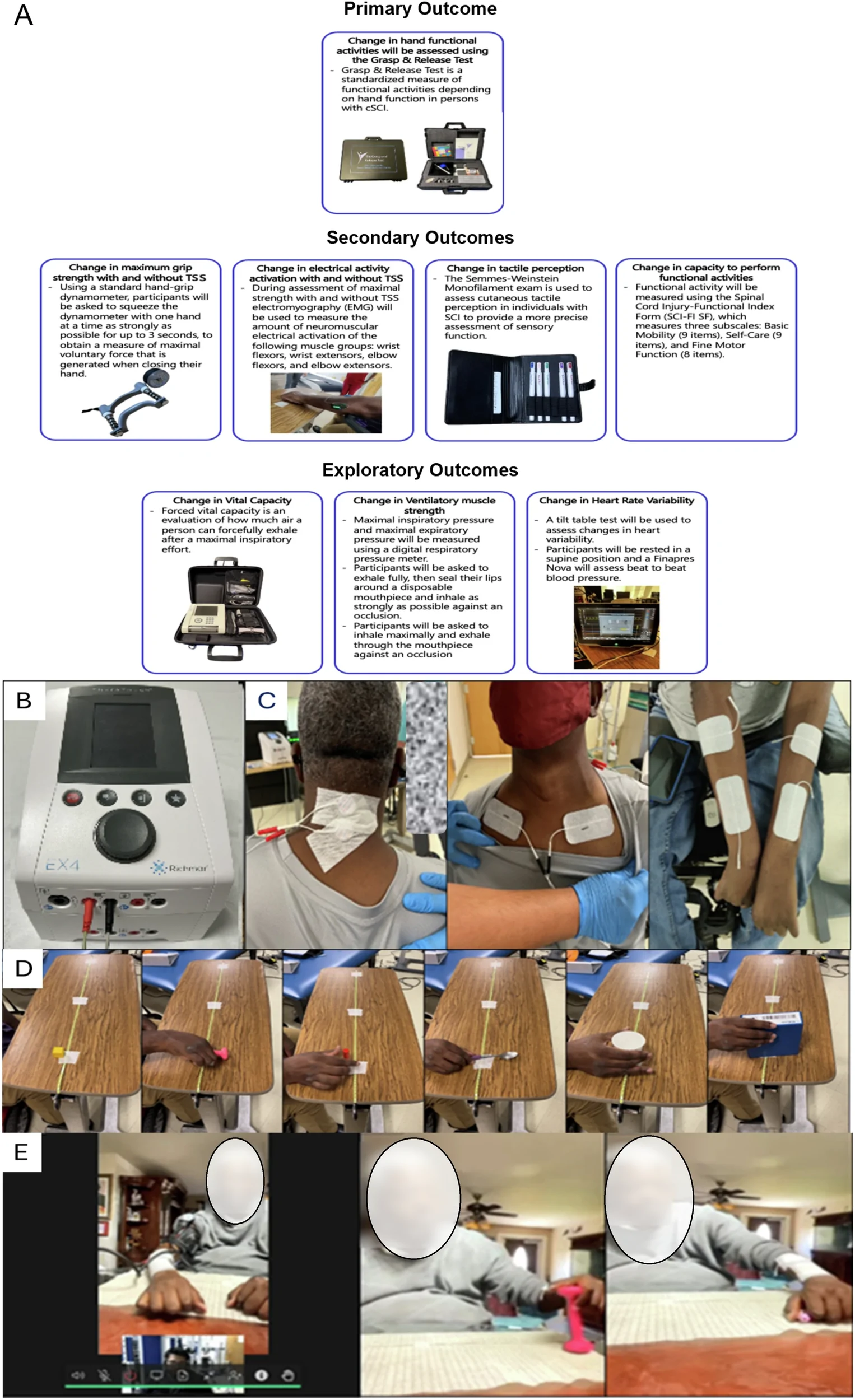
Study overview and intervention components. (**A**) Primary and secondary outcomes assessed from baseline through week 20. Primary: Grasp and Release Test (GRT); maximal grip strength without and with TSS; surface EMG amplitude without and with TSS; tactile perception. Secondary: capacity to perform functional activities; vital capacity; ventilatory muscle strength; and heart-rate variability (HRV). (**B**) Richmar EX-4 stimulator used to deliver TSS. (**C**) Electrode montage: TSS electrodes over the cervical spine and bilateral clavicles; neuromuscular electrical stimulation (NMES) electrodes placed bilaterally over wrist extensor muscles. (**D**) Grasp-and-release training sequence (block, 1-lb weight, peg, spoon, cup, cassette). (**E**) Telehealth-supervised session in which the participant performs grasp-and-release exercises during TSS. EMG, electromyography; TSS, transcutaneous spinal cord stimulation; NMES, neuromuscular electrical stimulation; HRV, heart-rate variability; GRT, Grasp and Release Test.

This is a standardized assessment of hand-dependent functional activities in individuals with incomplete tetraplegia [17, 20]. Participants will lift and transfer objects (Fig. 2) from lateral to medial positions and release them over a 60 cm distance. Six items (peg, spoon, block, 1 lb. weight, cylinder and videocassette) will be placed on a smooth table and allow the participants to choose their own grasping strategy. Successful completion requires either a pinch grip or palmar grasp with upright placement upon release. All trials will be video recorded for scoring; participant identity will be protected by excluding facial images, and recordings will not be used for non-research purposes without written consent.

**Safety and Feasibility of the trial** Adherence and compliance will be reported for each participant throughout the trial. Adherence will be evaluated via reporting the total number of missed sessions, the number of SCI participants who will be approached, reasons for non-participation, and burden of data collection on the participant and caregiver. Safety measurements will include cardiovascular-autonomic safety of the intervention. The number of episodes of developing autonomic dysreflexia or orthostatic hypotension will be reported throughout the trial [18].

#### Secondary outcomes

##### Maximum grip strength and electromyographic (EMG) activation

Maximum grip strength will be assessed using a standard hand-grip dynamometer. Participants will perform single-hand maximal voluntary contractions, sustaining each effort for 3 s starting with the right side and then followed by the left-hand grip. Up to 9 repetitions per side will be completed, with verbal encouragement and guidance provided to minimize compensatory movements. These are composed of three repetitions without TSS, three repetitions with TSS at 75% (submaximal motor threshold) and three repetitions with TSS at 100% (motor threshold) [11]. A 60-second rest will be given between attempts.

Surface electromyography (EMG) will be simultaneously recorded from the elbow flexors and extensors as well as wrist flexors and extensors to quantify neuromuscular activation patterns during each trial and determining the motor threshold for individual muscle group. Standard skin preparation (cleaning with alcohol and shaving if necessary) will be performed before electrodes placement to optimize signal quality. A biphasic current of 2 ms and a frequency of 1 Hz will be gradually ramped up from 1–40 mA to determine the motor threshold of each individual muscle group. The motor threshold is defined as 20 µv in EMG response for each specific muscle group. The highest current responsible for evoking 20 µv in the 4 muscle groups, bilaterally, will be used as participant’s motor threshold.

##### Semmes-Weinstein monofilament test for sensation

The Semmes-Weinstein Monofilament Exam is used to assess cutaneous tactile perception in individuals with SCI to provide more precise assessment of sensory function when considered in combination with the International Standards for Neurological Classification of Spinal Cord Injury (ISNCSCI) exam. Semmes-Weinstein Monofilaments are small, thin fibers attached to a handle that bend once a certain amount of force [ranging from 0.07–300 grams] is applied [21]. With eyes closed, participants will be asked whether they can feel a light touch from the monofilaments at standard locations on the pam and dorsum of the hand and fingers. Participants are given a score depending on the thinnest filament and t the smallest amount of force needed to perceive a tactile stimulus.

##### Spinal cord injury–functional index short form [SCI-FI SF]

The SCI–FI SF assesses functional activity capacity in individuals with SCI across three subscales expected to improve with TSS + BES + MP: Basic Mobility (9 items), Self-Care (9 items), and Fine Motor Function (8 items). Participants rate each item on a 4-point scale (1 = “with much difficulty” to 4 = “without any difficulty”). Subscale scores are summed and converted to standardized T-scores, where higher values indicate greater functional capacity. Tetraplegia-specific versions of the Self-Care and Fine Motor Function subscales will be used. The SCI–FI SF demonstrates excellent internal consistency and multi-site reliability [22].

#### Exploratory outcomes

##### Pulmonary function assessment

Pulmonary function will be evaluated using Forced Vital Capacity (FVC) [23], Maximal Inspiratory Pressure (MIP) [24], and Maximal Expiratory Pressure (MEP) [24] to quantify ventilatory capacity and respiratory muscle strength. Tests will be performed with participants seated, using a digital spirometer and respiratory pressure meter equipped with disposable mouthpieces and antiviral/antibacterial filters.

For FVC, participants will perform maximal inhalation followed by forceful exhalation into the spirometer for the device-specified duration. For MIP, participants will exhale fully, then inhale maximally against an occluded airway; for MEP, they will inhale fully, then exhale maximally against an occlusion. Each test will include at least three attempts to ensure maximal effort, with ≥ 60 s of rest between trials. The resulting values will be recorded for analysis. These standardized assessments provide quantitative measures of ventilatory muscle strength and pulmonary function in individuals with tetraplegia.

##### Autonomic-cardiovascular assessment

Autonomic function will be assessed with standardized procedures routinely used for persons with SCI in a research setting [25]. Heart-rate variability (HRV) provides an indirect index of the autonomic nervous system’s ability to adapt to physiological stressors. HRV will be analyzed off-line using LabChart software (AD Instruments) from data recorded with a 5-lead ECG. A continuous beat-by-beat blood pressure is also measured via a finger cuff after performing brachial calibration. The LabChart software has built-in module to calculate HRV from the recorded ECG data. Frequency domain indices (low frequency [LF] and high frequency HF]) will be calculated from ECG recordings during baseline, tilt, and recovery to characterize autonomic adaptability to changes in posture [26]. For simplicity, a lower LF:HF ratio suggests a higher parasympathetic activity, whereas a higher ratio suggests a dominance of the sympathetic activity [26].

Participants will rest quietly in the supine position for 5 min before the table is gradually raised to a 60° head-up tilt. Straps at the knees, hips, and chest will secure standing posture during tilt. The position will be maintained for up to 10 min unless systolic blood pressure falls ≥ 40 mm Hg below the resting baseline or the participant requests termination of the test due to the development of orthostatic symptoms, following previously reported standard procedures [25]. Testing will conclude with another 5-min supine recovery period while ECG and blood-pressure signals continue to be recorded.

##### Interventions

All intervention procedures, both onsite (Phase 1) and at home under telehealth supervision (Phase 2), will be performed with participants seated in their own wheelchairs. Participants and companions may ask questions and take rest breaks as needed.

##### Education and setup

Participants and caregivers will complete a 4-week education phase (2 weeks onsite, 2 weeks via telehealth) to train them on device setup (Richmar Ex4 Stimulator), operation, and safety for all stimulation and exercise components. Under supervision, caregivers will be trained to independently position the electrodes, adjust the current intensity, and operate the stimulators during home sessions. EX4 is widely used in clinical settings and commercially available with a friendly touch screen. The safety of the EX4 unit has been previously reported in a home clinical trial designed to stimulate denervated muscles in persons with SCI [27].

##### Intervention protocol

TSS will be applied at two midline spinal sites (C3–C4 and C6–C7) using circular 2 cm cathodes and 5 × 10 cm rectangular anodes that are placed bilaterally on the convex medial two-thirds of the clavicles. Clavicles allow for simple placement of the electrodes and immediate skin inspection against potential irritation. Stimulation will consist of rectangular biphasic (2 ms) pulses at 30 Hz, intensities titrated to produce mild, non-painful paresthesia without motor activation [11]. Training stimulation will be set at 75% of the established motor threshold to ensure integration of the descending supraspinal control during training [11]. Sham TSS will use identical electrode placement but 5 Hz, 0.1 ms pulses 10–15% below sensory threshold [9, 10, 11].

MP training will include unilateral and bilateral task-specific exercises emphasizing grasp-and-release control. Tasks will target lateral prehension (peg, 1 lb. weight, fork), palmar prehension (block, can, videotape), and bilateral coordination (typing, calculator keys, jar opening), individualized to participant goals (Table 1).

**Table 1:** Layout of blocks of training that will occur in each training session for both BES + TSS + MP and BES+sham TSS + MP groups.

BES + TSS + MP or BES+sham TSS + MP						
	Week 1			Week 2		
Block	Day 1	Day 2	Day 3	Day 1	Day 2	Day 3
2: Uni MP	L arm, lateral prehension	R arm, palmar prehension	L arm, palmar prehension	R arm, lateral prehension	Repeat week 1, day 1	Repeat week 1, day 2
3: BES	L arm	R arm	L arm	R arm		
4: Uni MP	R arm, lateral prehension	L arm, palmar prehension	R arm, palmar prehension	L arm, lateral prehension		
5: BES	R arm	L arm	R arm	L arm		
6: Bi MP	Bilateral tasks	Bilateral tasks	Bilateral tasks	Bilateral tasks		

All training blocks will be performed in the presence of TSS [group 1] or sham TSS [group 2]. Blocks of training will consist of unilateral massed practice exercises, BES, or bilateral massed practice exercises. Note that Block 1 [not shown in tables) will be used to set up the TSS, equipment for MP exercises, and BES. Each block will be approximately 10 min. All MP blocks will consist of practicing 3 different tasks for 3 min each, while BES blocks will consist of BES procedures as described previously. Participants will complete training sessions each day in the order described in the table above for the duration of the study. “Lateral” and “palmar” prehension refer to different kinds of gripping techniques that will be practiced in each session.

*MP*, massed practice; *BES*, blood flow restriction-enhanced neuromuscular electrical stimulation; *TSS*, transspinal spinal cord stimulation; *L*, left; *R*, right; *Uni*, unilateral; *Bi*, bilateral.

In the BES + TSS + MP group, participants will perform 16 weeks of training (3 sessions/week) focused on wrist-extensor strengthening under blood-flow restriction (BFR) combined with TSS. After measuring systolic BP, a 20.75 × 5.75 in cuff inflated to 30% above resting systolic pressure will be placed proximal to the elbow joint [17]. Surface NMES (20 Hz, 500 µs pulses) will be delivered via two carbon-film electrodes over the wrist extensors. The amplitude of the current (mA) will be gradually increased until functional wrist extension is achieved without eliciting extension of the fingers [17]. The NMES-BFR training will be synchronized with TSS to elicit three sets of 10 repetitions (5 s on/5 s off) per arm starting with the right arm. Eash set is followed by 2 min of rest after completely deflating the cuff to restore normal blood flow circulation in the trained arm.

Following familiarization, participants and caregivers will continue training sessions at home for 16 weeks using portable stimulator unit (Fig. 2), an automatic BP monitor, and manual BFR cuff. Telehealth supervision will replicate onsite procedures, with real-time feedback from study staff via secure video conferencing.

##### Statistical analysis

Continuous variables will be summarized as means ± standard deviations or, when appropriate, as medians with interquartile ranges; categorical variables as counts and proportions. In accordance with CONSORT guidelines, no baseline group comparisons will be conducted, as randomization ensures any differences occur by chance. However, baseline difference [BL2-BL1] will be considered to account for any gains during the 4-week education phase. All analyses will follow the intention-to-treat (ITT) principle, with participants analyzed in their assigned groups regardless of adherence. Data assumptions will be assessed, and transformations or non-parametric tests applied as needed.

Treatment effects of BES + TSS on the primary outcome (Grasp and Release Test at follow-up) will be analyzed using analysis of covariance (ANCOVA) including treatment group, baseline (BL2) score, time since injury, and injury severity as covariates. Inclusion of the baseline score, uncorrelated with treatment but highly correlated with outcome, increases statistical power without bias. A two-sided alpha of 0.05 will be used. Secondary outcomes will be evaluated using parallel ANCOVA models with identical covariates and alpha level (0.05).

For primary specific aim, effect sizes of the TSS + BES intervention compared to the sham TSS + BES group will also be calculated using the following equation.

$$	{{{\rm{Effect}}}}\; {{{\rm{size}}}}=[{{{\rm{Post}}}}\mbox{-}{{{\rm{intervention}}}}\; {{{\rm{Measurements}}}}{\mbox{-}} \\ 	 {{{\rm{Baseline}}}}\; {{{\rm{Measurements}}}}]/{{{\rm{Pooled}}}}\; {{{\rm{standard}}}}\; {{{\rm{deviation}}}}$$

Cohen’s D cut-off points will be used to determine the strength of the intervention [small effect ≥ 0.2, medium effect ≥ 0.5 and large effect ≥ 0.8].

Missing data will be handled using multiple imputations with chained equations, generating 25 imputed datasets via predictive mean matching under a missing at random assumption. Variables associated with missingness will be included in the imputation model. Complete-case sensitivity analyses assuming missing completely at random*)* will be performed to assess robustness. All statistical analyses will be conducted using SPSS software (IBM Corp., Armonk, NY, USA).

##### Sample size calculation

Our sample size is selected to provide sufficient power for testing the efficacy of BES + TSS on the grasp and release test. Additionally, statistical analyses are selected to provide the most power given the limited sample pool available. These power calculations are also applied to the other outcome variables. We propose a total sample size of 44, assuming 10% attrition during the study. The statistical power for the ANCOVA model depends on the mean difference, standard deviation, and variance explained by the covariates.

We, therefore, expect to observe a large effect in our proposed study. Additionally, as is common in pre-post treatment designs, we expect there to be a large correlation ( > 0.5) between pre- and post-measurements observed in our sample. With our proposed sample size, we will have 0.80 power to detect a Cohen’s d of 0.53 assuming 25% of the variance in outcome is explained by the covariates, corresponding to a medium effect. If we conservatively assume only 9% of the variance in the outcome is explained by the covariates, we will have a statistical power of 0.80 to detect a Cohen’s d of 0.65, which is between a medium (0.5) and large (0.8) effect. If we assume a 0.7 correlation between the pre- and post-treatment measurement (49% of the variance in the outcome is explained by the covariates), we will have a statistical power of 0.80 to detect a Cohen’s d of 0.35, which is between a small (0.1) and medium (0.5) effect. Given our ability to robustly find a medium effect size, we consider the sample size sufficient to accomplish the study goals.

## Discussion

TSS has been previously shown to safely and feasibly enhance upper-extremity function in individuals with tetraplegia [28]. We hereby propose a 20-week multimodal rehabilitation approach of synergistically apply TSS in conjunction with BES and MP training in persons with incomplete tetraplegia. The trial primarily targets home-training setting to reduce several of the barriers associated with lab-based interventions. Persons with tetraplegia typically experience inherent challenges related to available caregivers, dressing, transfer and transportation. These factors are likely to cause exhaustion, fatigue and reduce QOL. Therefore, a home-based multimodal approach may enhance adherence, compliance and reduce unnecessary travel time and associated costs of transportation [29]. Furthermore, adopting applications of telehealth videoconference communication may broaden the program to wide cohort with incomplete SCI, especially in rural areas [27, 29].

TSS is a noninvasive neuromodulation strategy that has been shown to enhance neuroplasticity and restore motor control after SCI [8, 10, 12, 28, 30]. The TSS restorative therapy is likely to amplify the motor function via posterior root muscle reflex [13]. Together with MP training, persons with SCI are likely to show improvement in both motor and sensory outcomes. A recent single-arm multicenter trial demonstrated the feasibility of Onward TSS stimulation in improvement of upper extremity function [31]. Each participant served as his own control and recognizable improvement in hand function were clearly documented following 8 weeks of training [31]. A subset of the original trial continued to show improvement in hand functions following 8 weeks of home-based training [32].

Effective rehabilitation strategies experience delay until they become available in clinical settings. Researchers and future clinical trials need to strive to bridge such research-clinical gap. For such purpose, a commercially available EX4 stimulator was used in the current trial for an off-shelf indication to deliver non-carrier frequency TSS [18]. Furthermore, the non-carrier frequency pulses previously demonstrated effective stimulation of the lumbosacral segments when TSS was combined with exoskeletal assisted walking in persons with SCI [33]. The successful use of non-carrier frequency TSS will ensure immediate translation to clinical settings without the need to go through extensive regulatory approvals. Today, we screened 13 participants, six completed the entire trial without a single adverse event, two are currently active and five screen failed for number of reasons. All participants can tolerate the non-carrier frequency TSS current and showed overtime decline in the amplitude of the current required to induce motor threshold.

The rationale of combining TSS with BFR exercise is geared towards improving upper extremity strength and hand function via enhancing both neuroplastic and muscular adaptations [10, 17, 34]. Previously, BES has been shown to increase wrist-extensor muscle size and strength [17]. Six weeks of BES resulted in enhancing hand function in persons with incomplete SCI [17]. However, the effect of BES was only limited to the wrist extensor muscles without targeting the major upper extremity muscle groups. Therefore, TSS is used to engage the proximal and distal muscle groups and to further augment upper extremity functions [10]. Improvement in sensory pathways may further augment motor improvement of the hand functions [35]. Therefore, we capture the changes in sensory outcomes as measured by Semmes-Weinstein test, a noninvasive test quantifying tactile threshold.

As an exploratory aim, effects on cardiovascular-autonomic and pulmonary functions are considered in the current proposal. Persons with incomplete SCI are at heightened risks of developing an array of autonomic dysfunctions including orthostatic intolerance and autonomic dysreflexia [36, 37]. Therefore, we hypothesize that the current intervention may enhance cardiovascular autonomic regulation, reflected by improved HRV and blood-pressure stability during orthostatic stress challenging-test [18, 30]. Strengthening autonomic control mechanisms may mitigate orthostatic hypotension and related cardiovascular risks. Feasibility testing demonstrated tolerance of repeated tilt-table assessments without orthostatic symptoms in persons with SCI. All test terminations occurred primarily due to dropping below the predefined resting systolic BP [25, 36]. Finally, our feasibility trial demonstrated the safety of TSS application on cardiovascular-autonomic function in a home-based setting [18].

The proximity of TSS application to C3-C5 spinal cord segments may lead to phrenic nerve activation and stimulation of the diaphragm muscle (i.e. primary inspiratory muscle). Therefore, it is possible to assume that TSS application may be accompanied by improvement in pulmonary functions in persons with incomplete tetraplegia. Furthermore, the combination of TSS, BES, and MP is anticipated to improve pulmonary function by facilitating respiratory muscle activation and promoting systemic neuroplastic changes [30]. Increases in FVC, MIP, and MEP would represent clinically meaningful gains, potentially reducing respiratory complications that drive morbidity in tetraplegia. However, given that the intervention does not directly target other major respiratory muscles, improvements may be modest and influenced by variability in baseline respiratory function.

### Limitations

Participant compliance depends heavily on caregiver availability; missed or delayed sessions occurred when caregivers are unavailable. It appears more logical to use brachial units to monitor BP during home-based training. However, caregivers will be given the option to choose between wrist or brachial units to measure BP for simplicity purpose. To minimize disruption, identifying a secondary caregiver during the education phase is recommended. Muscle spasms during data collection occasionally interfered with EMG signal stability and motor-threshold determination. The use of a manual instead of a digital dynamometer reduced grip-strength measurement precision, especially for values below 2 kg. Pulmonary testing may be limited by impaired trunk stability and volitional control in participants with high-level injuries. Participants may have trouble maintaining posture or sustaining consistent effort during FVC, MIP, and MEP trials, leading to variable results. Difficulty in maintaining an airtight mouth seal may also introduce other source of variability. Cardiovascular-autonomic assessments may be influenced by number of factors including blood-pressure modifying medications, caffeine intake, and incomplete bladder emptying. Finally, hand contractures and minor skin trauma limited electrode placement and occasionally prevented testing on the affected hand.

### Trial status

It is currently an active trial that has been open for enrollment since [03/23/2022] with anticipated completion date of [9/30/2026]. The study was approved by Richmond VA IRB since 03/23/2022 and R&D since [5/20/2022]. Recruitment started [6/15/2022] and will end [9/30/2026].

## Data Availability

Data will be shared by the lead contact author [Ashraf Gorgey (ashraf.gorgey@va.gov)] upon request and after obtaining necessary approval from the local research office.
